# Evolution of Phospholipase A2 in Bees and Flies

**DOI:** 10.1002/ece3.72385

**Published:** 2025-10-19

**Authors:** Siqi Yang, Minyu Wu, Ping Feng

**Affiliations:** ^1^ Key Laboratory of Ecology of Rare and Endangered Species and Environmental Protection (Guangxi Normal University), Ministry of Education Guilin Guangxi China; ^2^ The Chongzuo White‐Headed Langur Field Observation and Research Station of Guangxi Chongzuo Guangxi China; ^3^ Guangxi Key Laboratory of Rare and Endangered Animal Ecology Guangxi Normal University Guilin Guangxi China

**Keywords:** evolutionary analysis, insect venoms, phospholipase A2

## Abstract

Phospholipase A2 (PLA2) as a component of venom has been studied intensively in venomous snakes while little has been done in insects. In this study, we firstly identified *PLA2* among 36 Hymenoptera (*Apis* and *Bombus* genera) and 28 Diptera (*Drosophila* genus) by data mining on the known genomes. As a result, a total of 115 sequences were obtained, including 73 intact genes, 12 partial genes, and 30 pseudogenes. Most species have 1; 1 species from the *Drosophila* genus has 0, whereas both 1 species from the *Drosophila* genus and 9 species from the *Bombus* genus have two intact *PLA2* genes. Secondly, 4 previously published intact *PLA2* genes (1 from *Apis*, 2 from *Bombus*, and 1 from *Drosophila*) were added to the dataset of 73 intact genes and compared the evolutionary differences between the toxic group (*Apis* and *Bombus* genera) and non‐toxic group (*Drosophila* genus) by conducting selective pressure analyses and protein structure prediction. The results showed that *PLA2* from both Hymenoptera (*Apis* and *Bombus* genera) and Diptera (*Drosophila* genus) was under purifying selection. The evolutionary rate of the toxic group was larger than that of the non‐toxic group; furthermore, *PLA2* of *Bombus* species was subjected to accelerated evolution when compared with the other two groups. It may be attributed to the multiple venom injections of *Bombus* species, while in the *Apis* genus, the species has only one chance to inject, although both genera have stingers. In contrast, *Drosophila* has no stinger to inject and its *PLA2* has other functions, which may link with development. This study lays the foundation for understanding the different roles of *PLA2* in the genera *Apis*, *Bombus*, and *Drosophila*.

## Graphical abstract

This study investigated the evolution of the PLA2 gene in bees and flies and compared the evolutionary rate between them, which denotes the toxic group and the non‐toxic group. The results showed that PLA2 was under purifying selection and that an accelerated evolution of PLA2 occurred in the toxic group when compared with the non‐toxic group.

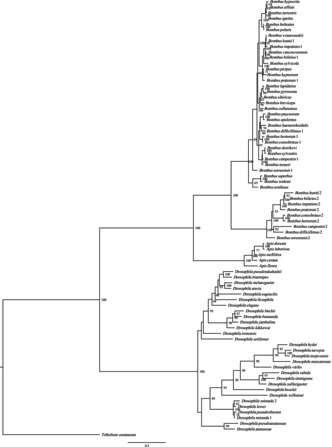

## 1 Introduction

The occurrence of venom is an important development during the evolutionary process of animals, especially insects (Barua and Mikheyev 2020). Animal venom systems are unparalleled biological systems to study molecular evolution (Zancolli and Casewell 2020). Bees can release venoms to defend themselves from attack (Liu et al. 2024). The primary compositions of bee venoms are proteins and peptides, of which melittin is the most abundant component, while phospholipase A2 (PLA2) has been characterized intensively (Gauldie et al. 1976; Argiolas and Pisano 1985; Six and Dennis 2000; Xin et al. 2009). PLA2 is rich in the venoms of animals (Valdez‐Cruz et al. 2007; Xin et al. 2009), and it can release lysophospholipids to combine with melittin, which increases the rate of red blood cell lysis (Watala and Kowalczyk 1990; Perez‐Riverol et al. 2019).

Insect venom phospholipases have been detected in many species of Hymenoptera such as bees and wasps (Perez‐Riverol et al. 2019). Apian PLA2 is the most well‐reported toxin from insect venoms (dos Santos‐Pinto et al. 2018) and was characterized as the main allergen in some species from *Apis* (King et al. 1976) and *Bombus* (Hoffman and Jacobson 1996; Hoffman et al. 2001) genera (Perez‐Riverol et al. 2019). The proportion of bee venom PLA2 in dry weight of honeybee venoms (HBV) and bumblebee venom can reach 12% (Jakob et al. 2017). Based on the chronology of discovery, the expressed sites of the body, differences in substrate specificity as well as physiological function, animal PLA2s can be divided into 16 groups (Groups I–XVI) and six subfamilies, such as secreted PLA2s (sPLA2s) (Groups I, II, III, V, IX, X, XI, XII, XIII, and XIV), cytoplasmic PLA2s (cPLA2s) (Group IV), and so on (Six and Dennis 2000; Dennis et al. 2011; Hrithik et al. 2023).

Among the various types of PLA2, secreted PLA2 (sPLA2) is the most common one in Hymenoptera (Kilaso et al. 2016; Perez‐Riverol et al. 2019), and group III sPLA2 is the main member of the sPLA2 family of insects, characterized by low molecular weight enzymes, a high degree of disulfide bonding, and dependence on Ca^2+^ (Kilaso et al. 2016). It can hydrolyze the sn‐2 ester bond of glycerophospholipids on the biological membrane in the presence of Ca^2+^, releasing free fatty acids and lysophospholipids, and mediating the destruction of cell membranes, the formation of pores, and necrosis (Perez‐Riverol et al. 2019). They are involved in a variety of cellular processes, which include phospholipid digestion and metabolism, host defense, and signal transduction (Kilaso et al. 2016). Snakes, bees, scorpions, and spiders all contain *PLA2* genes (snakes often possess 20–40 *PLA2* genes), and PLA2 can assist in prey digestion, intraspecific competition, and attack resistance (Fry et al. 2009). *PLA2* has been isolated from many sources, such as snake venom (Brunie et al. 1985; Ducancel et al. 1988), mammalian pancreas (Dijkstra et al. 1981), liver, etc. (Betzel et al. 1999), and it not only participates in immobilizing and killing prey in snakes but also involves many biological processes such as host defense and signal transduction in mammals (Dennis 1983; Lynch 2007). It is suggested that PLA2 from bees displays some homology to that from mammalian pancreas and snake venoms, and the structure of the anterior region as well as the activation mechanism of *PLA2* is different between mammals and reptiles (Kuchler et al. 1989); besides, mammalian and reptilian PLA2s comprise overlapping yet distinct sub‐families, which may contribute to the diversity of *PLA2* structure and function.

Venom systems are responsible for toxin production, storage, and injection (Walker et al. 2018). The diversification of venomous systems can be characterized by morphological features, such as skull, dentition, glands in reptiles (Fry et al. 2009), and mouthpart/non‐mouthpart structures, cuticular spines in insects (Walker et al. 2018). Some insects use mouthparts to inject venom into prey, and some employ mouthparts related structures (e.g., mandibles) to conquer and dissolve prey when hunting; other insects, such as some species from Hymenoptera, utilize non‐oral structures (e.g., sting or aculeus) to catch prey (Walker et al. 2018). Hymenoptera species (bees) are the best known venomous insects (Nakashima et al. 1995), and their ovipositor has formed a stinger for venom injection (Koludarov et al. 2023).

Insect venom has a variety of functions, such as subduing prey by some larvae and adults of Diptera or deterring predators by some bees (Walker et al. 2018). PLA2 is one of the major venom allergens that has an essential role in Hymenopteran insects' defense (Baumann et al. 2018). In the venomous insects, *Apis mellifera* and *Bombus ignitus* , *PLA2* sequences were identified (Kuchler et al. 1989; Xin et al. 2009). As a contrast, in the non‐venomous species, *Drosophila melanogaster* , *PLA2* was also identified and it was found throughout the process of development (Ryu et al. 2003).

It has been found that venom‐encoding genes/gene families are undergoing rapid evolution, such as PLA2 in snakes (Kini and Chan 1999; Zhang et al. 2015; Barua and Mikheyev 2020), *Hsp70* gene in spiders (Starrett and Waters 2007), and neurotoxin coding genes from scorpions (Zhang et al. 2015). The rapid evolution may be due to the attenuation of purifying (negative) selection, which will lead to relaxed selection or positive selection (Harpur and Zayed 2013; Walker 2020). Meanwhile, a well‐established notion states that the functionally important gene tends to be subject to strong purifying selection to maintain the function (Creighton and Darby 1989; Kozminsky‐Atias and Zilberberg 2012). This notion has been examined in fishes, birds, and mammals and found that animals with higher demand for energy metabolism experienced more selective constraint on mitochondrial protein–coding genes to keep the function stable for energy production (Shen et al. 2009; Sun et al. 2011).

Venom is a powerful weapon for immobilizing prey and deterring predators, and it is critical for Hymenoptera insects (Fry et al. 2009). Thus, whether *PLA2* of Hymenoptera (*Apis* and *Bombus* genera) will be subjected to strong selective constraint due to the need to keep its function stable to kill the prey or rapid evolution as the PLA2 in snakes is uncertain. In contrast, in Dipteran (*Drosophila* genus) insects, which have no venom (Ryu et al. 2003; Stanley 2006), whether the PLA2 will be under different selective pressure remains to be seen. To solve these questions, we collected the *PLA2* sequences of Hymenoptera species, as well as *PLA2*s of Dipteran (*Drosophila* genus), representative of *PLA2*s from the toxic group and non‐toxic group, to investigate the evolution of *PLA2* in insects from these orders and compared their evolutionary trajectory differences between these two groups. This study focuses on the systematic analysis of *PLA2* genes in the genera of *Apis*, *Bombus*, and *Drosophila*, and will reveal the differentiation of *PLA2* evolutionary trends between Hymenoptera and Dipteran.

## 2 Materials and Methods

### 2.1 Data Acquisition

Genomes of the species from genera *Apis*, *Bombus*, and *Drosophila* were collected and downloaded from NCBI (https://www.ncbi.nlm.nih.gov/). The *PLA2* sequences of *Apis mellifera* (accession number: NM_001011614.1), *Bombus ignites* (FJ768908.1), *B. hypocrite* (KF214771), and *Drosophila melanogaster* (AY219032.1) were downloaded as query sequences for gene identification.

### 2.2 Identification of *PLA2* Genes

We used the published insect *PLA2* genes as query sequences and searched for *PLA2* in 64 currently available insect genomes using TblastN (Feng and Zhao 2013), with an E‐value cutoff setting at 1E‐10 (Jiao et al. 2018). To identify exons in each *PLA2* gene, scaffolds containing *PLA2* were downloaded and then the Blast 2 was conducted between each exon and scaffolds (Feng and Zhao 2013). All exons were assembled using ClustalX (Thompson et al. 1997) and compared with published *PLA2* from closely related species. The newly identified *PLA2*s were categorized into three categories: intact genes, partial genes, and pseudogenes. Intact genes are those with full‐length coding sequences and correct start and stop codons; partial genes refer to those with truncated open reading frames due to incomplete genome sequencing; and pseudogenes are genes with frameshift mutations (Jiao et al. 2019).

### 2.3 Sequence Alignment and Phylogenetic Reconstruction

In this section, the intact *PLA2* sequences from newly identified and downloaded from NCBI were imported into the MEGA 11 software (Tamura et al. 2021) and translated into amino acid sequences, then the resulting sequences were aligned and translated back to nucleotide sequences. The nucleotide sequence alignment was subsequently used for selective pressure analysis, and the phylogenetic tree of *PLA2* was reconstructed, with *PLA2* from *Tribolium castaneum* (FJ768722.1) used as an outgroup. NJ tree and ML tree were simply reconstructed by using MEGA 11, while the Bayesian inference (BI) tree was reconstructed by PhyloSuite (Zhang et al. 2020; Xiang et al. 2023), which integrated many programs such as MrBayes (Ling et al. 2016), MAFFT program (Rozewicki et al. 2019), and ModelFinder (Kalyaanamoorthy et al. 2017). Specifically, first, the collected data was imported into PhyloSuite, and then multiple sequence comparisons were performed using MAFFT to improve the accuracy of gene sequence comparisons. Second, sequences were concatenated together, and optimal partitioning and evolution models were selected for the dataset using ModelFinder. Finally, the phylogenetic tree was reconstructed using MrBayes, which was run for 5,000,000 generations with 1000 as the default value. The operation can be terminated when the ASDSF value is below 0.01 (Xiang et al. 2023).

### 2.4 Selective Pressure Analyses

Selective pressures acting on *PLA2* were analyzed through using PAML 4 (Yang 2007), with the phylogenetic tree as a guide tree, and the ratio of synonymous substitution rate to non‐synonymous substitution rate can indicate the selective pressure acting on protein‐coding genes. When *ω* = 1, it is under neutral evolution; *ω* < 1 suggests negative or purifying selection; *ω* > 1 indicates positive selection (Yang 2007). Firstly, model 0 (free ωs) was conducted by allowing a free ω to test the overall selective pressure of *PLA2*, and model 0 (*ω* = 1), which fixed ω to 1, was also conducted. Subsequently, these two models were compared to determine which model fits the data better. Secondly, we further used site models to detect the possibly positively selected sites through conducting a comparison of three pairs of models (Dasmeh et al. 2014). The first pair being M1a (near neutral) and M2a (positive selection), M1a assigned two kinds of sites: conserved sites and neutral sites. In the neutral site, the rate of synonymous substitution is equal to the rate of non‐synonymous substitution, whereas, at the conserved site, the rate of non‐synonymous substitution is zero (Nielsen and Yang 1998), and in the M1a model, the rate of synonymous substitution is constant and the rate of non‐synonymous substitution is variable (Nielsen and Yang 1998). The M2a model is a positive selection model, and it is an extension of the M1a model (Nielsen and Yang 1998). The second pair is M7(*β*) and M8(*β* & *ω*); the M7 model assumes that all sites are 0 < *ω* < 1 and β‐distributed, while M8 is based on M7 with the addition of positively selected sites (*ω* > 1) (Shi et al. 2003). The third pair is M8a (*β* & *ω* = 1) and M8 (*β* & *ω*), and M8a is similar to M8, but ω is fixed to Dasmeh et al. (2014). M1a, M7, and M8a are null hypotheses, while M2a and M8 are alternative hypotheses. Amino acid sites subjected to positive selection were examined by the Bayes Empirical Bayes (BEB) method (Yang et al. 2005), and a site would be recorded when its corresponding posterior probability (PP) was larger than or equal to 95% (Dong et al. 2021). It is proposed that the residues distributed in the protein surface tend to accumulate variations, and they are subjected to positive selection, while the core residues are conserved (Sunagar, Fry, et al. 2013; Sunagar, Jackson, et al. 2013), and some studies suggested that such tactic was employed by toxins from Hymenoptera (Brust et al. 2013; Ruder et al. 2013; Baumann et al. 2018). Thus, in this study, we will examine this hypothesis.

To test whether the positively selected sites detected on pressure analyses were on the surface of the protein, we used SWISS‐MODEL (Waterhouse et al. 2018) to model the protein structure and check the locations of positively selected sites. Thirdly, to investigate whether *PLA2* evolves differently between the toxic group and the non‐toxic group, a two‐ratio model analysis which allows branches leading to *Drosophila* to be ω_1_ while othersω_2_ was conducted; a three‐ratio model analysis assigning branches leading to *Drosophila*, *Apis*, and *Bombus* to be ω_1_, ω_2_, and ω_3_, respectively, was also carried out. Fourthly, model comparison was executed.

## 3 Results

### 3.1 *PLA2* Identification and Phylogenetic Tree Analysis

Genomes of 64 insect species from 3 genera were downloaded from NCBI, including 4 from *Apis*, 32 from *Bombus*, and 28 from *Drosophila*. Genome coverage ranged from 15.0× to 370.0× (but genome coverage data were not available for *Drosophila busckii* , *D. melanogaster* , and *D. jambulina*), and contig N50 ranged from 24.9 kb to 34201.6 kb. The detailed information on the genome of species used in this study was listed in Table S1. The search of these 64 publicly available insect genomes yielded 115 *PLA2* genes, including 73 intact genes, 12 partial genes, and 30 pseudogenes (Table 1). The length for the *PLA2* sequence of each genus is 504–516 bp for *Apis*, 543 bp for *Bombus*, and 561–570 bp for *Drosophila*. Gene sequences are listed in Supplementary Text S1.

**TABLE 1:** Number of *PLA2* genes identified in this study.

	Genus	Species	Number of *PLA2* gene			
			Intact	Partial	Pseudo	Total
Toxic group	*Apis*	*A. cerana*	1	0	0	1
		*A. dorsata*	1	0	0	1
		*A. florea*	1	0	0	1
		*A. laboriosa*	1	0	0	1
	*Bombus*	*B.vosnesenskii*	1	0	1	2
		*B. polaris*	1	1	1	3
		*B. difficillimus*	2	0	0	2
		*B. confusus*	1	0	1	2
		*B. opulentus*	1	0	1	2
		*B. skorikovi*	1	1	1	3
		*B. superbus*	1	0	3	4
		*B. bifarius*	2	0	0	2
		*B. soroeensis*	2	0	1	3
		*B. consobrinus*	2	0	1	3
		*B. picipes*	1	0	2	3
		*B. sibiricus*	1	1	0	2
		*B. cullumanus*	1	0	1	2
		*B. balteatus*	1	1	1	3
		*B. haemorrhoidalis*	1	1	0	2
		*B. breviceps*	1	1	0	2
		*B. pyrosoma*	1	0	1	2
		*B. turneri*	1	1	1	3
		*B. sylvicola*	1	0	1	2
		*B. huntii*	2	0	0	2
		*B. hortorum*	2	0	1	3
		*B. pascuorum*	1	0	1	2
		*B. campestris*	2	0	1	3
		*B. hypnorum*	1	1	1	3
		*B. waltoni*	1	1	2	4
		*B. sylvestris*	1	1	1	3
		*B. pratorum*	2	0	0	2
		*B. lapidarius*	1	1	1	3
		*B. impatiens*	2	0	0	2
		*B. affinis*	1	0	2	3
		*B. vancouverensis*	1	0	1	2
		*B. terrestris*	1	0	2	3
Non‐toxic group	*Drosophila*	*D. busckii*	1	0	0	1
		*D. sulfurigaster*	1	0	0	1
		*D. virilis*	1	0	0	1
		*D. hydei*	1	0	0	1
		*D. immigrans*	1	0	0	1
		*D. mercatorum*	1	0	0	1
		*D. rubida*	1	0	0	1
		*D. grimshawi*	0	1	0	1
		*D. willistoni*	1	0	0	1
		*D. setifemur*	1	0	0	1
		*D. pseudotakahashii*	1	0	0	1
		*D. pseudoobscura*	1	0	0	1
		*D. pseudoananassae*	1	0	0	1
		*D. navojoa*	1	0	0	1
		*D. mojavensis*	1	0	0	1
		*D. miranda*	2	0	0	2
		*D. lowei*	1	0	0	1
		*D. kikkawai*	1	0	0	1
		*D. jambulina*	1	0	0	1
		*D. ironensis*	1	0	0	1
		*D. ficusphila*	1	0	0	1
		*D. eugracilis*	1	0	0	1
		*D. erecta*	1	0	0	1
		*D. elegans*	1	0	0	1
		*D. bunnanda*	1	0	0	1
		*D. birchii*	1	0	0	1
		*D. biarmipes*	1	0	0	1
		*D. ananassae*	1	0	0	1

Most species have 1 intact *PLA2* gene; 1 species from the *Drosophila* genus ( *D. grimshawi* ) has none; 1 species from the *Drosophila* genus (D. miranda) and 9 species from the *Bombus* genus ( *B. impatiens* , *B. pratorum* , *B. campestris* , *B. huntii* , *B. difficillimus* , *B. bifarius, B. consobrinus, B. hortorum*, and *B. soroeensis* ) have two intact *PLA2* genes, and in order to distinguish them, we named them 1 and 2. The number of *PLA2* genes varies among genera, with 1–2 intact genes, 1–2 partial genes, and 1–2 pseudogenes in *Bombus* species; 1 intact gene, 0 partial genes, and 0 pseudogenes in *Apis* species; while 0–2 intact genes, 0–1 partial genes, and 0 pseudogenes in *Drosophila* species (Table 1). In the following analysis, the *PLA2* sequences of *Apis mellifera* , *Bombus ignitus* , *B. hypocrita* , and *Drosophila melanogaster* downloaded directly from GenBank were also added to the intact genes identified in this study. Thus, in sum, 77 intact *PLA2* genes from 39 Hymenoptera (*Apis* and *Bombus* genera) and 29 Diptera (*Drosophila* genus) were used.

**FIGURE 1:**
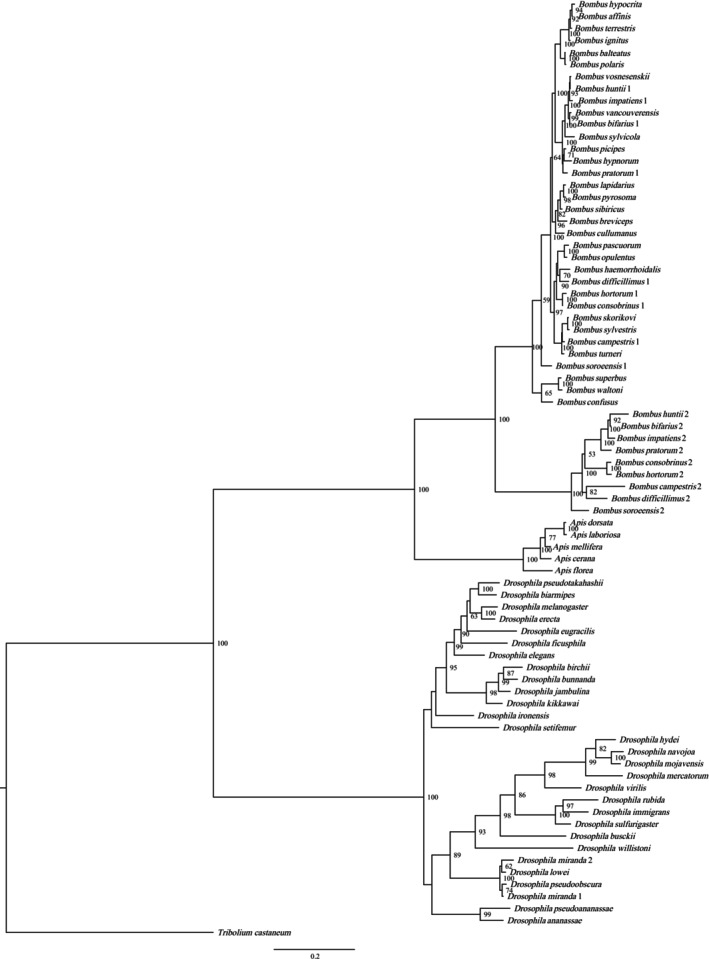
BI tree of intact *PLA2* genes from three genera.

The result of the phylogenetic tree showed that, as a whole, the BI tree has higher posterior probabilities than that of the ML tree and NJ tree (see Figure S1 for ML and Figure S2 for NJ tree). In the BI tree, *PLA2* genes of the genera *Apis*, *Bombus*, and *Drosophila* have high similarity within the genus while having relatively low similarity among genera; thus, the phylogenetic tree showed that they clustered according to the genera (Figure 1). In *Bombus* species, *PLA2* genes that have two copies cluster into two groups, with one copy gathering with that of another single *PLA2* gene, while another copy clusters with the rest (Figure 1), indicating that the ancestor gene of *PLA2* duplicated before species differentiation.

**FIGURE 2:**
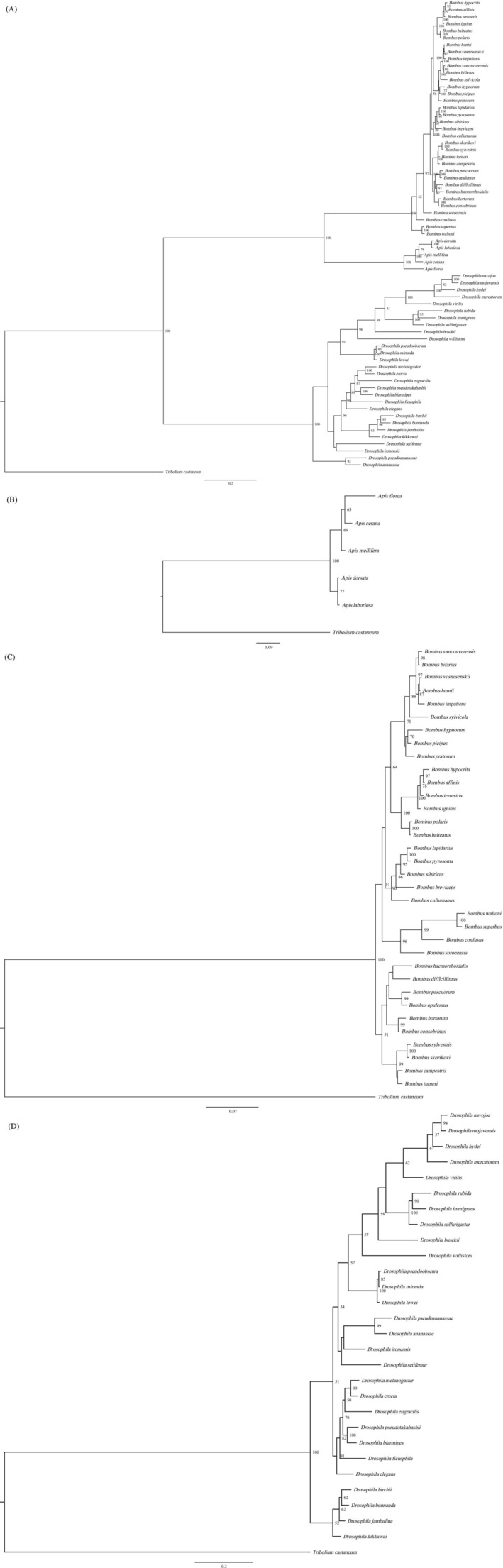
BI tree of intact *PLA2* from three genera (without copy2) (A), *Apis* (B), *Bombus* (without copy2) (C), and *Drosophila* (without copy2) (D). *Tribolium castaneum* phospholipase A2 was used as an outgroup.

For the subsequent analyses, BI tree for all the intact *PLA2* without copy2, BI tree for *Apis*, *Bombus* (without copy2), and *Drosophila* (without copy2) were also reconstructed, respectively. The resultant trees were shown in Figure 2A–D.

### 3.2 Selection Pressure Analyses

**FIGURE 3:**
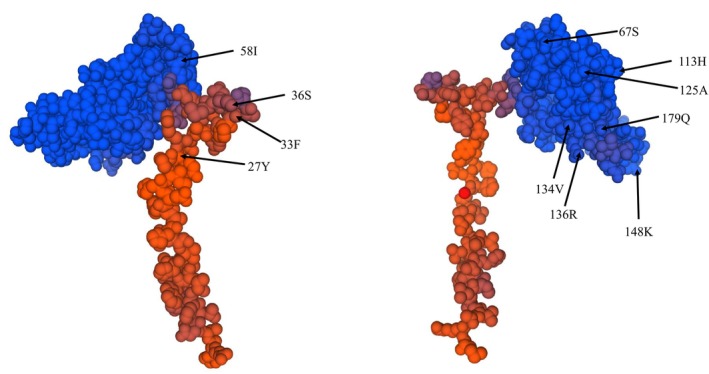
Locations of positively selected sites detected in selective pressure analyses.

In order to explore the selection pressure acting on *PLA2* genes of the dataset mentioned above, we performed multiple modeling analyses on each dataset by using the corresponding phylogenetic tree in Figure 2, that is, Figure 2A–D was used as the guide tree for selective pressures analyses for all the intact *PLA2* of three genera without copy2, *PLA2* sequences of *Apis*, *PLA2* sequences of *Bombus* (without copy2), and *PLA2* sequences of *Drosophila*, respectively. The result showed that *PLA2* consisting of all the intact genes without copy2 was under strong purifying selection (*ω* = 0.12, *p* = 0), and no positively selected site was detected (Table 2). Selective pressure analyses showed that *PLA2* of *Drosophila*, *Apis*, and *Bombus* were subjected to purifying selection, with ω being 0.08, 0.26, and 0.54, respectively, indicating that *PLA2* of *Apis* and *Bombus* may be subject to accelerated evolution. In addition, three (58 I, 67 S, 148 K) and 11 (27 Y, 33 F, 36 S, 58 I, 67 S, 113 H, 125 A, 134 V, 136 R, 148 K, 179 Q) positively selected sites were detected in Model2a and Model8 of *Bombus* (Table 3), respectively, suggesting that positive selection signatures existed in these genes. After conducting protein structure prediction, we found that all the 11 positively selected sites were on the surface of PLA2. The result was shown in Figure 3 (Table 4).

**TABLE 2:** Selective pressure analyses of the intact *PLA2* gene sequences without copy2.

Model	Parameter estimates	2*dL* for model comparison	*p*	Positively selected sites
Model 0 (*ω*)	*ω* = 0.12	1507.50 (M0 (*ω*) versus M0 (*ω* = 1))	**0**	None
Model 0 (*ω* = 1)	*ω* = 1			
Model 1a (nearly neutral)	*p*_0_ = 0.88, *ω*_0_ = 0.09 *p*_1_ = 0.12, *ω*_1_ = 1.00	0 (M2a versus M1a)	1.00	None
Model 2a (positive selection)	*p*_0_ = 0.88, *ω*_0_ = 0.09 *p*_1_ = 0.01, *ω*_1_ = 1.00 *p*_2_ = 0.11, *ω*_2_ = 1.00			
Model 7 (beta)	*p* = 0.44, *q* = 2.56	0 (M8 versus M7)	1.00	None
Model 8 (beta & *ω*)	*p*_0_ = 1.00, *p* = 0.44 *q* = 2.56 *p*_1_ = 0, *ω* = 2.76			
Model 8a (beta & *ω* = 1)	*p*_0_ = 1.00, *p* = 0.44 *q* = 2.56 *p*_1_ = 0, *ω* = 1.00	0.06 (M8 versus M8a)	0.81	None

*p* < 0.05 is marked in bold.

**TABLE 3:** Selective pressure analyses of *PLA2* gene sequences (without copy2) from the species of genera *Apis*, *Bombus*, and *Drosophila*, respectively.

Model	Parameter estimates	2*dL* for model comparison	*p*	Positively selected sites
*Apis*				
Model 0 (*ω*)	*ω* = 0.26	40.52 (M0 (*ω*) versus M0 (*ω* = 1))	**1.95E‐10**	None
Model 0 (*ω* = 1)	*ω* = 1			
Model 1a (nearly neutral)	*p*_0_ = 0.70, *ω*_0_ = 0 *p*_1_ = 0.30, *ω*_1_ = 1.00	0 (M2a vs. M1a)	1.00	None
Model 2a (positive selection)	*p*_0_ = 0.70, *ω*_0_ = 0 *p*_1_ = 0.21, *ω*_1_ = 1.00 *p*_2_ = 0.09, *ω*_2_ = 1.00			
Model 7 (beta)	*p* = 0.01, *q* = 0.03	0.50 (M8 vs. M7)	0.78	None
Model 8 (beta & *ω*)	*p*_0_ = 0.80, *p* = 0 *q* = 0.06 *p*_1_ = 0.20, *ω* = 1.00			
Model 8a (beta & *ω* = 1)	*p*_0_ = 0.70, *p* = 0 *q* = 1.52 *p*_1_ = 0.30, *ω* = 1.00	0 (M8 vs. M8a)	1.00	None
*Bombus*				
Model 0 (*ω*)	*ω* = 0.54	25.58 (M0 (*ω*) vs. M0 (*ω* = 1))	**4.24E‐07**	None
Model 0 (*ω* = 1)	*ω* = 1			
Model 1a (nearly neutral)	*p*_0_ = 0.66, *ω*_0_ = 0.03 *p*_1_ = 0.34, *ω*_1_ = 1.00	28.26 (M2a versus M1a)	**7.30E‐07**	58 I‐0.986* 67 S‐1.000** 148 K‐0.966*
Model 2a (positive selection)	*p*_0_ = 0.64, *ω*_0_ = 0.03 *p*_1_ = 0.28, *ω*_1_ = 1.00 *p*_2_ = 0.08, *ω*_2_ = 3.21			
Model 7 (beta)	*p* = 0.02, *q* = 0.02	28.12 (M8 vs. M7)	**7.83E‐07**	27 Y‐0.955* 33 F‐0.952* 36 S‐0.964* 58 I‐0.997** 67 S‐1.000** 113 H‐0.989* 125 A‐0.954* 134 V‐0.991** 136 R‐0.972* 148 K‐0.994** 179 Q‐0.990**
Model 8 (beta & *ω*)	*p*_0_ = 0.93, *p* = 0.02 *q* = 0.03 *p*_1_ = 0.07, *ω* = 3.49			
Model 8a (beta & *ω* = 1)	*p*_0_ = 0.66, *p* = 2.78, *q* = 99.00, *p*_1_ = 0.34, *ω* = 1.00	27.08 (M8 versus M8a)	**1.95E‐07**	None
*Drosophila*				
Model 0 (*ω*)	*ω* = 0.08	2867.22 (M0 (*ω*) vs. M0 (*ω* = 1))	**0**	None
Model 0 (*ω* = 1)	*ω* = 1			
Model 1a (nearly neutral)	*p*_0_ = 0.86, *ω*_0_ = 0.05 *p*_1_ = 0.14, *ω*_1_ = 1.00	0 (M2a versus M1a)	1.00	None
Model 2a (positive selection)	*p*_0_ = 0.86, *ω*_0_ = 0.05 *p*_1_ = 0.14, *ω*_1_ = 1.00 *p*_2_ = 0, *ω*_2_ = 54.35			
Model 7 (beta)	*p* = 0.24, *q* = 2.11	0 (M8 versus M7)	1.00	None
Model 8 (beta & *ω*)	*p*_0_ = 1.00, *p* = 0.24 *q* = 2.11 *p*_1_ = 0, *ω* = 26.72			
Model 8a (beta & *ω* = 1)	*p*_0_ = 1.00, *p* = 0.24 *q* = 2.11 *p*_1_ = 0, *ω* = 1.00	0 (M8 vs. M8a)	1.00	None

* *p* > 0.95.

** *p* > 0.99.

*p* < 0.05 is marked in bold.

**TABLE 4:** Likelihood ratio tests of selective pressures on *PLA2* (without copy2) from insects of three genera (*Apis*, *Bombus*, and *Drosophila*).

Models	*ω*(*d*_N_/*d*_S_)	2dL	Model comparison	*p*
Data set: *PLA2* sequences of 5 *Apis* species, 34 *Bombus* species and 28 *Drosophila* species				
A. All branches have the same *ω*	*ω* = 0.12	169.8	B vs. A	8.18E‐39
B. All *Apis* and *Bombus* branches have *ω*_2_, and other branches have *ω*_1_	*ω*_1_ = 0.08 *ω*_2_ = 0.36			
*C. bombus* branches have *ω*_3_, *Apis* branches have *ω*_2_, and *Drosophila* branches have *ω*_1_	*ω*_1_ = 0.08 *ω*_2_ = 0.25 *ω*_3_ = 0.42	4.98	C vs. B	0.03

To test whether the accelerated evolution exists in *PLA2* of toxic group (*Apis* or *Bombus* or *Apis* + *Bombus*), two‐ratio and three‐ratio model analyses were conducted. A two‐ratio model analysis showed that branches leading to *Drosophila* have *ω*_1_ = 0.08 while branches leading to *Apis* and *Bombus* have ω_2_ = 0.36, and this model fits the data better (*p* = 8.18E‐39) than does one‐ratio model which assigns one *ω* to all the branches, indicating that *PLA2* evolved differently between the toxic group and non‐toxic group, and the toxic group was subjected to an accelerated evolution. A three‐ratio model analysis which allows branches leading to *Drosophila*, *Apis*, and *Bombus* to have *ω*_1_, *ω*_2_, and *ω*_3_ respectively showed that *ω*_1_ = 0.08, *ω*_2_ = 0.25, and *ω*_3_ = 0.42. A further comparison between two‐ratio and three‐ratio revealed that the three‐ratio was better suited to the dataset (*p* = 0.03), suggesting that the evolutionary rate in *PLA2* of *Bombus* was significantly different from that of *Apis*, and *PLA2* of *Bombus* had experienced accelerated evolution. All the results were listed in Table 4.

## 4 Discussion

In a previous study, Baumann et al. (2018) investigated the selective pressures acting on the six kinds of allergens (phospholipase A1, phospholipase A2, acid phosphatase, hyaluronidase, serine protease, and antigen 5) in Hymenoptera and compared the types of selection across these six groups of allergens using coding sequences downloaded from GenBank. In this study, we focus on the phospholipase A2 in Hymenoptera (*Apis* and *Bombus*) and Diptera (*Drosophila*), which denote the toxic group and non‐toxic group, to investigate the evolution of *PLA2* in insects from these three genera and explore whether the evolutionary patterns were different between the toxic and non‐toxic groups and within toxic groups by using the gene sequences identified from genomes of the species from the *Apis*, *Bombus*, and *Drosophila* genera. Species in *Bombus* have one or two intact *PLA2* genes, which is consistent with the suggestion that many genes have one or more copies in almost all species of bumblebees (Sun et al. 2021). In addition, all the *Apis* species and most *Drosophila* species investigated in this study have a single‐copy intact *PLA2* gene, while a few species of *Bombus* have two copies. One of the *PLA2* copies of *Bombus* clustered together, which is assumed to have originated from gene duplication and will become pseudogenes in the future. Just as it is suggested that gene duplication usually leads to an increase in gene dosage (Schuster‐Böckler et al. 2010; Zhou et al. 2011), the duplicated genes are mutated to separate them from the parental genes, and to a certain extent, they become pseudogenes and are no longer expressed (Zhang 2003). In addition, Fry et al. (2009) had proposed that during the recruitment of proteins as animal venoms, the adaptive evolution of toxins is often enhanced by extensive gene duplication once they are recruited, and then duplication of toxin genes may be selectively favored, leading to higher toxin doses. Therefore, the gene duplication of *PLA2* genes in some *Bombus* species may be attributed to the need to accumulate toxin dosage for faster gland venom replenishment.

As to the total number of *PLA2* genes in the *Bombus* genus is greater than that of the *Apis* genus. It is probably due to differences in venom dosage between *Apis* and *Bombus* species, which result from venom injections. The stings of species from the *Apis* genus have barbs, and only one injection of venom will take its life, while the stings of *Bombus* genus species do not have barbs, and multiple venom injections can be performed by them (Hoffman and Jacobson 1984). In *Drosophila*, one species ( *D. grimshawi* ) has 0 intact *PLA2* genes, while another species ( *D. miranda* ) has two intact *PLA2* genes, which may be due to the poor sequencing quality in such regions.

Selective pressure analyses revealed that *PLA2* was under purifying selection, no matter in the dataset consisting of species from *Apis* + *Bombus + Drosophila* or the dataset composed of each genus. This is consistent with the previous study which suggested that strong purifying selection had acted on publicly available hymenopteran *PLA2* (Baumann et al. 2018) and that its selective pressure was different from that of the venomous genes of advanced snakes which were reported to experience rapid evolution and positive selection (Sunagar, Jackson, et al. 2013; Dutertre et al. 2014). Considering Hymenoptera diversified about 281 million years ago (Peters et al. 2017), purifying selection may have governed the *PLA2* for a long time, suggesting that PLA2 played a critical role in such a group of insects.

Compared to the evolution of *PLA2* between Diptera and Hymenoptera, which denoted a non‐toxic group and a toxic group, we found that the evolution of *PLA2* in the non‐toxic group was significantly different from that of the toxic group, with *ω =* 0.36 in Hymenoptera while *ω =* 0.09 in Diptera, suggesting that *PLA2* in Hymenoptera had been subjected to accelerated evolution. In *Bombus*, we even detected 11 positively selected sites under model 8, and further location examination suggested that these 11 positively selected sites were on the surface of *Bombus* PLA2, in accord with the previous finding which proposed that the residues exposed on the surface were subjected to positive selection (Sunagar, Fry, et al. 2013; Sunagar, Jackson, et al. 2013; Sunagar, Undheim, et al. 2013). Moreover, the variation accumulations on the protein surface are beneficial to changing the physical and chemical properties of the protein, which causes neofunctionalisation (Baumann et al. 2018), and the positive selection on the surface of PLA2 protein is assumed to cause neofunctionalisation. Together, these results may indicate that PLA2 in the toxic group is prone to vary to adapt to the complex and volatile environment. Although some codons on the surface are subjected to positive selection, most sites are under purifying selection to keep the function conservative.

As to the anatomic feature, it is reported that, in Apidae, the venom apparatus is undergoing degeneration (Kerr and De Lello 1962); that is, from *Psithyrus* to *Bombus* to *Meliponini* (“stingless bees”), the venom gland goes from well‐developed to intermediate to nearly completely disappeared, and the venom reservoir may also be degraded or disappeared (Kerr and De Lello 1962; Robertson 1968). In addition, honeybees (*Apis* genus) can only perform venom injections once during their lifetime (Hoffman and Jacobson 1984), and the degeneration and infrequent use of venom may be one of the reasons why the *PLA2* in Hymenoptera is under purifying selection.

Considering the function of PLA2 in the insects used in this study, *PLA2* expresses throughout the developmental process of *Drosophila*, and in adults, it expresses in bodies and heads (Ryu et al. 2003). The function of *Drosophila PLA2* is not well understood, and it is hypothesized to be related to *Drosophila* growth and development (Ryu et al. 2003). In *Bombus*, Gao et al. (2013) found that *PLA2* genes were highly expressed in the venomous gland, ovary, and midgut of *Bombus hypocrita* , and that the tendency is first increasing and then decreasing with the increase of age; while Xin et al. (2009) revealed that in *Bombus ignites*, *PLA2* was venom gland‐specific expressed, and *PLA2* was further detected in venom sac and secreted venom. In honeybee, *PLA2* was isolated from the venom glands (Kuchler et al. 1989). The differences in expressed sites of *PLA2* among *Drosophila*, *Bombus*, and *Apis* indicated the different biological functions, and strong purifying selection on *Drosophila* suggested the important role of PLA2 in promoting growth and development.

Finally, PLA2 is one of the hymenopteran allergens, of which most functional activity is not known and only some basic functions have been described (Baumann et al. 2018). This study provides insight into the evolution of *PLA2* in insects, and helps understand the different roles of *PLA2* in the genera *Apis*, *Bombus*, and *Drosophila*.

## 5 Conclusions

In this study, we analyzed the evolution of *PLA2* in the genera *Apis*, *Bombus*, and *Drosophila*, and compared the differentiation between the toxic group and the non‐toxic group. The results showed that *PLA2* was subjected to strong purifying selection, and compared with the non‐toxic group, *PLA2* in the toxic group was under accelerated evolution. *PLA2* in some species of *Bombus* has two copies, which may relate to the dosage demand or the multiple uses of their stings. The positively selected sites in *Bombus*‐PLA2 are associated with their positions on the protein surface, which facilitate their response to the variable environment.

## Author Contributions

**Siqi Yang:** data curation (equal), investigation (equal), methodology (equal), writing – original draft (equal). **Minyu Wu:** data curation (equal), formal analysis (equal). **Ping Feng:** formal analysis (equal), funding acquisition (equal), investigation (equal), writing – review and editing (equal).

## Conflicts of Interest

The authors declare no conflicts of interest.

## Supporting information


**Figure S1:** ML tree for *PLA2* genes from three genera.
**Figure S2:** NJ tree for *PLA2* genes from three genera.

**Table S1:** The detailed information on genome of species used in this study.

**Text S1:** All the intact *PLA2* gene sequences used in this study.

## Data Availability

All the intact PLA2 gene sequences used in this study are deposited in Text S1.
