# Supplementary material for: Evolution of Phospholipase A2 in Bees and Flies
Source: Ecol Evol. 2025 Oct 19;15(10):e72385. doi: 10.1002/ece3.72385 (PMC12535799; doi:10.1002/ece3.72385)
Supplement: Supplementary file 1 — Figure S1: ML tree for PLA2 genes from three genera. Figure S2: NJ tree for PLA2 genes from three genera. [file ECE3-15-e72385-s002.docx]

**Supplementary Figure S1** ML tree for *PLA2* gene from three genera

**Supplementary Figure S2** NJ tree for *PLA2* gene from three genera.
