# Supplementary material for: Evolution of Phospholipase A2 in Bees and Flies
Source: Ecol Evol. 2025 Oct 19;15(10):e72385. doi: 10.1002/ece3.72385 (PMC12535799; doi:10.1002/ece3.72385)
Supplement: Supplementary file 2 — Table S1: The detailed information on genome of species used in this study. [file ECE3-15-e72385-s001.docx]

**Supplementary Table 1** The detailed information on genome of species used in this study.

| **Genus** | **Species** | **Submitted GenBank assembly** | **Contig N50 (Mb)** | **Genome coverage (x)** |
| --- | --- | --- | --- | --- |
| *Apis* | *A. cerana* | GCA_029169275.1 | 13.39 | 87.8 |
|  | *A. dorsata* | GCA_009792835.1 | 0.03 | 37.3 |
|  | *A. florea* | GCA_000184785.2 | 0.02 | 20.5 |
|  | *A. laboriosa* | GCA_014066325.1 | 0.30 | 304.0 |
| *Bombus* | *B.vosnesenskii* | GCA_011952255.1 | 2.73 | 125.0 |
|  | *B.* *polaris* | GCA_014737335.1 | 0.43 | 100.0 |
|  | *B. difficillimus* | GCA_014737525.1 | 0.34 | 100.0 |
|  | *B. confusus* | GCA_014737475.1 | 0.41 | 100.0 |
|  | *B. opulentus* | GCA_014737405.1 | 0.73 | 100.0 |
|  | *B. skorikovi* | GCA_014737355.1 | 0.49 | 100.0 |
|  | *B. superbus* | GCA_014737385.1 | 0.99 | 100.0 |
|  | *B. bifarius* | GCA_011952205.1 | 2.20 | 125.0 |
|  | *B. soroeensis* | GCA_014737365.1 | 0.58 | 100.0 |
|  | *B. consobrinus* | GCA_014737455.1 | 0.71 | 100.0 |
|  | *B. picipes* | GCA_014737485.1 | 0.38 | 100.0 |
|  | *B. sibiricus* | GCA_014737505.1 | 0.56 | 100.0 |
|  | *B. cullumanus* | GCA_014737535.1 | 1.13 | 100.0 |
|  | *B. balteatus* | GCA_019201815.1 | 8.60 | 35.0 |
|  | *B. haemorrhoidalis* | GCA_014825975.1 | 0.87 | 100.0 |
|  | *B. breviceps* | GCA_014825925.1 | 1.04 | 100.0 |
|  | *B. pyrosoma* | GCA_014825855.1 | 0.78 | 100.0 |
|  | *B. turneri* | GCA_014825825.1 | 0.44 | 100.0 |
|  | *B. sylvicola* | GCA_019677175.1 | 3.02 | 30.0 |
|  | *B. huntii* | GCA_024542735.1 | 9.27 | 83.0 |
|  | *B. hortorum* | GCA_905332935.1 | 10.58 | 82.0 |
|  | *B. pascuorum* | GCA_905332965.1 | 8.97 | 28.0 |
|  | *B. campestris* | GCA_905333015.3 | 6.17 | 83.0 |
|  | *B. hypnorum* | GCA_911387925.2 | 11.59 | 33.0 |
|  | *B. waltoni* | GCA_014737395.1 | 1.15 | 100.0 |
|  | *B. sylvestris* | GCA_911622165.2 | 5.86 | 79.0 |
|  | *B. pratorum* | GCA_930367275.1 | 8.53 | 60.0 |
|  | *B. lapidarius* | GCA_936014575.1 | 2.31 | 15.0 |
|  | *B. impatiens* | GCA_000188095.4 | 0.06 | 127.0 |
|  | *B. affinis* | GCA_024516045.2 | 3.12 | 18.0 |
|  | *B. vancouverensis* | GCA_011952275.1 | 3.06 | 125.0 |
|  | *B. terrestris* | GCA_910591885.2 | 6.78 | 57.0 |
| *Drosophila* | *D. busckii* | GCA_011750605.1 | 1.00 | - |
|  | *D. sulfurigaster* | GCA_023558435.1 | 33.39 | 43.0 |
|  | *D. virilis* | GCA_003285735.2 | 8.70 | 100.0 |
|  | *D. hydei* | GCA_003285905.2 | 3.37 | 120.0 |
|  | *D. immigrans* | GCA_018153375.1 | 9.65 | 156.4 |
|  | *D. mercatorum* | GCA_961210405.1 | 22.67 | 79.0 |
|  | *D. rubida* | GCA_021223945.1 | 0.11 | 198.0 |
|  | *D. grimshawi* | GCA_018153295.1 | 23.29 | 92.8 |
|  | *D. willistoni* | GCA_018902025.2 | 9.08 | 150.0 |
|  | *D. setifemur* | GCA_021224005.1 | 0.07 | 179.0 |
|  | *D. pseudotakahashii* | GCA_021223935.1 | 0.04 | 149.0 |
|  | *D. pseudoobscura* | GCA_009870125.2 | 30.71 | 280.0 |
|  | *D. pseudoananassae* | GCA_018153035.1 | 7.39 | 110.5 |
|  | *D. navojoa* | GCA_001654015.2 | 0.03 | 81.0 |
|  | *D. mojavensis* | GCA_018153725.1 | 24.88 | 120.4 |
|  | *D. miranda* | GCA_003369915.2 | 11.98 | 100.0 |
|  | *D. lowei* | GCA_008121275.1 | 5.04 | 50.0 |
|  | *D. kikkawai* | GCA_018152535.1 | 21.81 | 113.0 |
|  | *D. jambulina* | GCA_018152175.1 | 24.62 | - |
|  | *D. ironensis* | GCA_021223825.1 | 0.17 | 84.0 |
|  | *D. ficusphila* | GCA_018152265.1 | 9.96 | 96.7 |
|  | *D. eugracilis* | GCA_018153835.1 | 2.29 | 118.0 |
|  | *D. erecta* | GCA_003286155.2 | 22.15 | 190.0 |
|  | *D. elegans* | GCA_018152505.1 | 21.93 | 108.2 |
|  | *D. bunnanda* | GCA_021223745.1 | 0.14 | 125.0 |
|  | *D. birchii* | GCA_008042755.1 | 0.21 | 36.0 |
|  | *D. biarmipes* | GCA_025231255.1 | 26.92 | 100.0 |
|  | *D. ananassae* | GCA_017639315.2 | 26.43 | 370.0 |
